# Birth of a Language in the Backlands of Brazil

**DOI:** 10.1111/cogs.70159

**Published:** 2025-12-28

**Authors:** Anderson Almeida‐Silva, Remo Nitschke, Fernando Valls Yoshida, Vitor Nóbrega, Shigeru Miyagawa

**Affiliations:** ^1^ Department of Linguistics Federal University of Pernambuco; ^2^ Institute for the Interdisciplinary Study of Language Evolution University of Zurich; ^3^ Department of Linguistics University of Arizona; ^4^ Department of Linguistics University of São Paulo; ^5^ Institute of Bioscience University of São Paulo; ^6^ Department of Linguistics Massachusetts Institute of Technology; ^7^ Research Center for Society 5.0 Seikei University

**Keywords:** Critical period, Emergent sign language, Homesign, Language acquisition, Language evolution, Sign languages

## Abstract

It is assumed that in order to acquire a language, children must be exposed to a language during the critical period, which generally lasts until puberty. Here, we report on Cena, an emergent sign language that has developed among a small group of deaf people in an isolated town in the state of Piauí, Brazil. Starting three generations ago, it has developed into a fully functioning communicative system with all characteristics of a typical human language even though Cena developed in a linguistic vacuum. What makes Cena interesting is that we are reasonably certain that Cena had no external input from the national sign language, Libras, or any other language during its formation. Cena challenges the assumption that to acquire the first language, the child must be exposed to a fully developed language. It developed from homesigns to an emergent sign language that is used for all aspects of village life. Cena also lends credence to the interactional model of language acquisition, which considers the interactions between the child and the caregivers to be the crucial element. The nativist model of language acquisition, which assumes a universal system underlying language, also plays a part. Through interaction, what arose is a system with characteristics essential to all human language.

## 1 Introduction

Emerging sign languages (or emergent sign languages) are new sign languages created by deaf signers without a prior signed or spoken language model (Meir, Sandler, Padden, & Aronoff, 2012). The basis for emergent sign languages are assumed to be homesigns, systems of signs created by congenitally deaf children born to hearing parents who have no knowledge of sign language (Begby, 2017; Botha, 2007; Goldin‐Meadow, 2005; Goldin‐Meadow & Feldman, 1977; Hill, Lillo‐Martin, & Wood, 2018; Richie, Yang, & Coppola, 2014). Earlier research on homesigns shows that once a sufficient number of such signers appear in a community, and they begin to interact with each other, a shared sign language may emerge, which is used as a medium of communication in a variety of social settings. Emergent sign languages may be broadly categorized as village sign languages, which arise within the context of a single village or small contiguous community, or deaf community sign languages, which arise in contexts where deaf children from different places are brought together (Meir et al., 2012).

Emerging village sign languages tend to arise when there is a higher‐than‐normal incidence of deafness in a village or small community. These signer communities are usually small ranging between a few signers to numbers in the low hundreds (Vos & Zeshan, 2012). In recent years, the number of reported village sign languages has increased significantly, and it is likely that there are many more than are currently known (Sandler, Padden, & Aronoff, 2022; Vos & Zeshan, 2012). The kinds of environments under which village sign languages arise can vary, from mid‐size towns such as Kafr Qasem with a population of roughly 21,000 to small villages such as is the case we will describe here. Transmission of the language happens within the village and familial context, and the language is used primarily within the village.

Emerging deaf community sign languages arise when signers of various backgrounds are brought together in schools, clubs, or other locations (Meir et al., 2012). Transmission of the language usually happens in those locations, but the language may be used within a larger community, such as a national deaf community.

Examples of emergent village sign languages include the Al‐Sayyid Bedouin Sign Language (ABSL) and Kafr Qasem Sign Language of Israel (Kastner, Meir, Sandler, & Dachkovsky, 2014; Sandler, Aronoff, Padden, & Meir, 2014; Sandler, Meir, Padden, & Aronoff, 2005), Kata Kolok of Bali (Branson, Miller, & Marsaja, 1996), Ka'apor Sign Language (Kakumasu, 1968) of Brazil, Adamorobe Sign Language of Ghana (Nyst, 2007), Bhan Khor of Thailand (Nonaka, 2007), and Central Taurus Sign Language of Turkey (Ergin, 2017; Ergin, Meir, Aran, Padden, & Jackendoff, 2018). Examples of emergent community sign languages include the Nicaraguan Sign Language (Morgan & Kegl, 2006) and Israeli Sign Language (Sandler et al., 2022).

In this paper, we report on Cena, an emergent village sign language that developed in the community of Várzea Queimada (VQ) in the state of Piauí, Brazil. We present interviews with community members that describe the history of the language emergence and development of Cena, and we describe linguistic features of Cena based on a multilingual corpus of the language. We suggest that the existence of Cena and other emergent sign languages challenges commonly held beliefs about language acquisition. We propose that Cena constitutes a particularly interesting case of language emergence, as we can show that it arose in complete isolation of any ambient sign language for the first ∼70 years of its history.

## 2 Theories of language acquisition

The existence of emerging sign languages and homesigns challenges the general assumption that in order to acquire a language, the child must be exposed to a fully developed language, spoken or signed. In the usual course of language acquisition, a child is exposed to the language of their caregivers and environment, and this naturally appears to trigger linguistic ability in the child.

There are two main contemporary theories on how children acquire language: the interactional model and the nativist model. It is generally assumed that in order for language acquisition to take place, one must be exposed to the target language at an early age. The interactional theory considers interaction with caregivers and others around the child to be the essential factor in language acquisition (Bruner, 1961; Levinson, 2019; Tomasello, 1992). For the nativist approach, the early exposure activates a biologically endowed predisposition for language learning that forms the foundation for the acquisition of any language, commonly called “universal grammar” (Chomsky, 1957, 1999; Pinker, 1994).

The nativist theory and the interactional theory are typically put up against each other, as they approach the origin of language differently. From the interactionist perspective, language evolved because of our communicative and interactive nature (Levinson, 2019), while the nativist approach assumes language to have arisen separately (Berwick & Chomsky, 2017). We suggest that both explanations are necessary to explain what we see in homesigns and later emergent sign languages. Infants crave interaction and that activates the inner linguistic resource present in all humans to acquire a language as a native language.

A prominent example of the interactionist approach is Levinson's Interactional Engine Hypothesis. Levinson assumes that humans are “endowed” with this Engine, which provides the underlying ability to communicate. Levinson assumes this Engine is separate from language, and its essential components are found in all the major clades of the primates.

The driving force of Levinson's theory is that “[h]uman social life is held together by intense social interaction. It has been estimated that humans spend up to 30% of waking hours in such interaction” (Levinson, 2016). The skills of interaction are culturally transmitted from the caregiver to the child as part of acquisition of communication and language (Levinson, 2019). For homesigns, the hearing caregiver engages in intense interaction with the deaf child through hand gestures and other means. Although the caregiver's gestures do not resemble language in failing to have any consistent lexical, morphological, or syntactic properties (Flaherty, Hunsicker, & Goldin‐Meadow, 2021; Goldin‐Meadow & Mylander, 1984; Goldin‐Meadow & Mylander, 1998; Goldin‐Meadow, Goodrich, Sauer, & Iverson, 2007; Goldin‐Meadow, Mylander, & Butcher, 1995), the interactions invite the child to respond. The signs produced by the child reflect many of the essential properties of human language lexically, morphologically, and syntactically.

The nativist theory contends that as humans, we have a biologically endowed predisposition for language learning, what Chomsky and others refer to as universal grammar (Chomsky, 1999; Berwick & Chomsky, 2017). Central to the nativist theory is the idea of poverty of stimulus (POS), which addresses how children can acquire complex grammatical structures even when the input is incomplete or ambiguous (Baker, 2001; Chomsky, 1965, 1986; O'Grady, 1997; Pinker, 1994). Children often demonstrate knowledge of rich and complex grammatical structures that are not found in the input data. This includes the implicit knowledge of hierarchical structures, which are not a part of the input in any overt fashion. The nativist approach assumes that children are able to learn a language despite the incomplete input since the biologically endowed capacity already contains a system that, when triggered, generates the essential properties of language. Homesigns and the emergent sign languages they develop into can be considered extreme cases of POS. The deaf children did not have any input from a mature language, spoken or signed. Yet, homesigners created linguistic systems that exhibit essential properties of language lexically, morphologically, and syntactically. Once enough signers are present, the system may stabilize into a multi‐generational language. What made the activation of this system possible was the intense interactions the caregivers had with the child. Hence, both the interactional theory and the nativist theory may be leveraged to explain homesigns and by proxy emergent sign languages like Cena that arose from homesigns.

## 3 Other emergent sign languages

The best‐known case of an emergent sign language is that of Nicaraguan Sign Language (NSL). In this case, Nicaraguan homesigners were initially isolated in families with hearing parents, and their signs were reported to be rudimentary and holistic, unlike the composite signs we see in more developed sign systems (Kegl & Iwata, 1989; Morgan & Kegl, 2006). However, in 1977, a school for the deaf was founded in Managua and quickly expanded to 100 deaf students by 1979 (Senghas & Coppola, 2001). While the teaching itself focused on lip‐reading in Spanish, the children were allowed to sign outside the classroom. They began to converge on a shared vocabulary of signs and consistent ways to express these signs, collectively creating a stable language, particularly in social settings like the school bus and the playground. This process led to the emergence of the Nicaraguan Sign Language (Kegl, Senghas, & Coppola, 1999; Senghas, 2003). NSL can be classified as a deaf community sign language, as it emerged from a situation where deaf individuals from various backgrounds were brought together and later dispersed again (Sandler et al., 2022). The emergence and transmission of NSL includes individuals of various ages and regional/familial backgrounds. Cena, on the other hand, arose within a village community and is used by signers who live in close contact in one contiguous community. As these differences could influence the course of language acquisition and emergence, it appears prudent to look to a language that arose in similar circumstances as Cena.

A better comparison to Cena is another well‐known case of emergent sign language: ABSL (Sandler et al., 2014). ABSL, like Cena, is a village sign language, meaning it arose within a contiguous village community. In both cases, there is a higher‐than‐average incidence of deafness in the village (around 3.5% for both cases) (Meir, Israel, Sandler, Padden, & Aronoff, 2012; Sandler et al., 2014) and some degree of ethnic or geographic separation that keeps the community comparatively isolated. While we have found some differences between ABSL and Cena, as seen in the section on lexical stability, their overall situation and development share a lot of similarities. Both Cena and ABSL are used in all aspects of village life by deaf and hearing signers (Almeida‐Silva & Nevins, 2020; Pereira, 2013; Sandler et al., 2014).

While ABSL and NSL differ in the histories of their emergence, they have in common that homesigners were brought together, be it through schooling or by being born in the same village. Once homesigners are brought together, they seem to converge on a shared system that can be passed on to subsequent generations as an emergent sign language. These sign languages emerged among the small community of deaf signers who were still within their critical period for language acquisition. The critical period is initiated by biological mechanisms that transform the brain into a state of plasticity that makes it possible for input to restructure the circuit for learning, a state that lasts until about puberty (Hensch, 2004; Knudsen, 2004; Lenneberg, 1967; Werker & Hensch, 2015; Wiesel & Hubel, 1963). Once the critical period is closed off, learning a language can no longer take place naturally and effortlessly, instead requiring substantial investment of time and effort by the learner.

## 4 A description of Cena

**Fig. 1:**
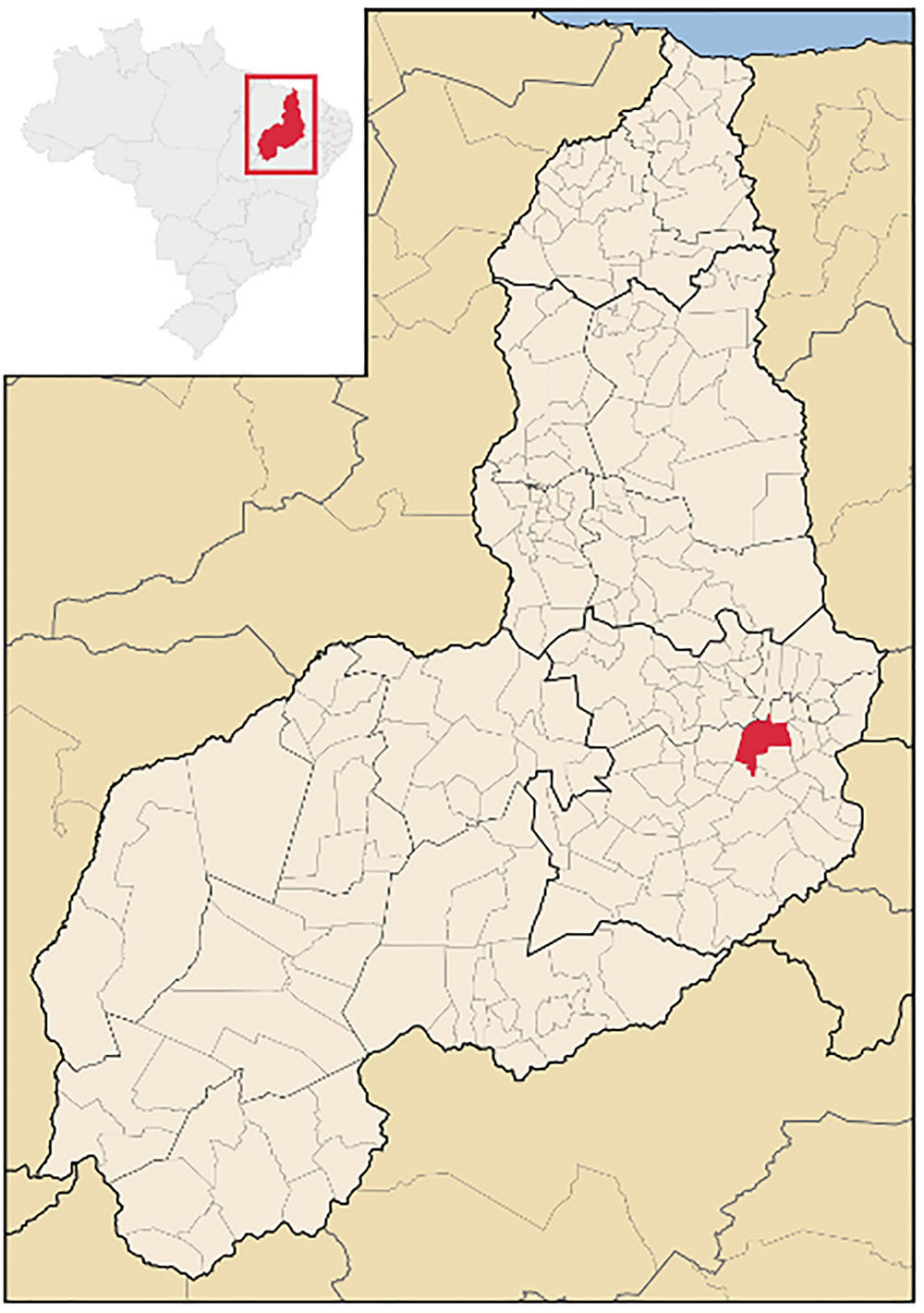
Jaicós municipality, in which the Várzea Queimada (VQ) community lies, Piauí/Brazil. Image created by Raphael Lorenzeto de Abreu (CC BY 2.5), source: Wikimedia.

Here, we report on Cena, an emergent village sign language that developed in the community of VQ, a village in the town of Jaicós, in the Southeastern portion of the state of Piauí, Brazil (Fig. 1). The community includes 900 individuals, of whom 34 are documented to be congenitally deaf (Almeida‐Silva & Nevins, 2020). The earliest documented deaf individual in the community was born in 1932, and at the time of writing, 33 of the 34 documented deaf village members are still alive. Most live in the village with a fluctuating number of around 10 who live in neighboring villages.

Cena displays many core features typical of human language, such as stable lexicon, linguistic productivity, and complex grammatical structure. We will present examples of these in subsequent sections. The community considers Cena its language, and it fills this role in all types of social contexts. The language is used in everyday interactions between signers as seamless as spoken conversations between speakers. Cena is used like any other language, ranging from simple conversations to complex stories that go beyond the here and now. Cena signers use Cena to tell stories, relay recipes, gossip, and explore complex concepts such as politics, as we can see from the excerpt below (1). Note that double dashes (//) indicate a prosodic break:

(1) Maura: TODAY DAWN SOW‐SEEDS FINISH // FEW FINISH
  “Today, very early in the morning (dawn), I planted the last seeds. There were a few of them, we are out of them now.”

In VQ, all members of the deaf community use Cena as their first language, while hearing members of the village exhibit varying levels of fluency. The deaf community is well integrated, and both deaf and hearing individuals frequently communicate with one another in Cena (Pereira, 2013). While the exact number of deaf signers is known (34), the exact number of hearing signers is currently unknown, but a large number display at least some degree of fluency. Deaf signers in the village use Cena for their daily interactions with other deaf signers as well as with hearing signers. Deaf signers who live in the neighboring villages tend to visit for cultural and religious events during which they communicate in Cena as well.

There exists some schooling for the deaf community in the form of night classes at the local school. This is a recent development. Many of the students are adult signers who dropped out of school in their younger years when schooling for the deaf was not yet established in the village. Unfortunately, the languages of instructions are mostly written in Portuguese and some Libras (the national SL of Brazil), both of which are unfamiliar languages to the signers (Franco, Fortes‐Lustosa, & Araújo, 2022). Most deaf signers are *not* bilingual between Cena and Libras, and teachers have reported a rejection of Libras by the signers in the educational environment (Almeida‐Silva & Nevins, 2020).

The beginnings of Cena can be traced back to 1932, with the birth of the first deaf individual in the community (Almeida‐Silva et al., 2023). Over the past 92 years, four generations of deaf children have been born in VQ, with the youngest generation ranging from 16 to 18 years of age.

What is unique about Cena is that we have a documented history of the language and its deaf signer community, from the earliest member born in 1932 to the multi‐generations of signers living today, and we have good evidence that the language arose in complete linguistic isolation (Pereira, 2013). From this documentation of first‐ and second‐hand accounts of members of the community, we know that Cena developed without any external spoken‐ or sign‐language influence. The signers did not have access to the spoken language of their community —namely, Brazilian Portuguese— because of deafness, and they only came into contact with Libras in 1998. Because Cena had been well established by that time, we see no meaningful borrowing from Libras in Cena today, as can be confirmed via the Cena–Libras dictionary (Almeida‐Silva et al., 2023). Hence, Cena has been largely unaffected by language contact throughout its history, although like any language, it continues to change as new speakers become part of the community.

## 5 Data and data collection

We now turn to the linguistic data from Cena. These data were initially collected by a team led by Anderson Almeida‐Silva with the goal of constructing a dictionary of the language based on authentic, spontaneous usage. The dictionary was organized and edited by Anderson Almeida‐Silva, who is an experienced sign language linguist and has been involved with deaf communities in Brazil for the past 24 years. Anderson Almeida‐Silva has been working with the Cena community since 2017 and is fluent in Cena signs. All the data collection was under the permission of the Brazilian National Ethical Committee (*Plataforma Brasil*—Ministry of Health), registered with the authorization number CAAE–26198719.4.0000.0121. In order to assemble a dictionary, the team first collected a dataset of naturalistic Cena data. This dataset includes various types of data, such as: (i) naturalistic conversations (e.g., dialogues); (ii) narrative retelling using video stimuli (e.g., Haifa clips—Sandler et al., 2005; pear history) or image prompts; (iii) lexical signs; and (iv) local stories (e.g., monologues). The dataset comprises more than 100 h of recording from the 24 deaf individuals still living in the community (ages ranging from 18 to 72 years old). This dataset was then used to assemble the recently released Cena dictionary (Almeida‐Silva et al., 2023). In this dictionary, 247 signs are listed, each with a concrete usage example drawn from the dataset, a Libras (Brazilian Sign Language) translation, and glossing in Portuguese. The Portuguese glossing and Libras translations were compiled by researchers familiar with Cena and Libras. The dictionary exclusively uses examples drawn from real utterances for the Cena entries. It is currently only available as a physical copy; a digital version is in preparation at the time of writing.

While the dictionary itself provides a valuable resource for the community, it also presents a glossed and translated corpus of the language. Every usage example is fully glossed and translated into both Libras and Brazilian Portuguese. This gives us a multilingual Cena–Libras–Portuguese parallel corpus based largely on naturalistic utterances. All language‐specific results presented in this paper are based on this corpus. Excluded from this are interviews we conducted with community members in order to assess the history of Cena. These are featured in the penultimate section of this paper.

## 6 Results

We will now report on our findings that show that Cena has a number of essential properties found in spoken and signed languages. We will highlight three properties, all typical characteristics found in human language: (i) lexical stability, (ii) the ability to combine elements to compose new signs, and (iii) grammatically complex expressions. We focus on these because we have collected a substantial amount of data for each, allowing us to reasonably conclude that they are well established in Cena.

### 6.1 Lexical stability

A critical component of language is a stable set of lexical items that are used consistently across different contexts. These lexical items do not refer to a specific instance of an object or person but rather denote a type of entity, such as *cow, house*, and *parent*. Goldin‐Meadow et al. (2007, 1995) observed that, in the case of homesigns, when parents attempted to communicate with the deaf child, their gestures were chaotic in nature, changing in shape from context to context. In contrast, once the deaf child established a sign for an object, it was used consistently across a variety of contexts, indicating acquisition of stable words similar to standard language (Singleton, Morford, & Goldin‐Meadow, 1993). Sandler et al. note that ABSL, which is the best studied of the emergent village sign languages, exhibits a high degree of inter‐signer variability in the way signs for the same lexical items are produced (Sandler, Aronoff, Meir, & Padden, 2011). They report that different versions of signs are distributed over different families, a phenomenon they call *familylect* (Sandler et al., 2014).

Although some lexical variation is also found across family units in VQ (Almeida‐Silva & Nevins, 2020), Cena exhibits a high degree of lexical stability. Signs are generally consistent across signers, which has enabled the creation of an extensive dictionary of Cena signs, where both the meaning and form of signs remain stable across users (Almeida‐Silva et al., 2023). The current dictionary contains 247 signs, which represent a subset of the full range of signs used in Cena. One possible explanation for the high degree of lexical stability may be the small size of the community. The VQ deaf community consists of only 34 individuals, with 24 residing consistently within the bounds of the village, whereas the Al‐Sayyid deaf community had approximately 130 members in 2012 (Kisch, 2012). Moreover, the VQ deaf community is distributed across 15 families. The small number of deaf individuals per family may hinder the development of distinct *familylects*, instead promoting lexical stability across the entire community of signers.

### 6.2 Linguistic productivity: Compounds

**Fig. 2:**
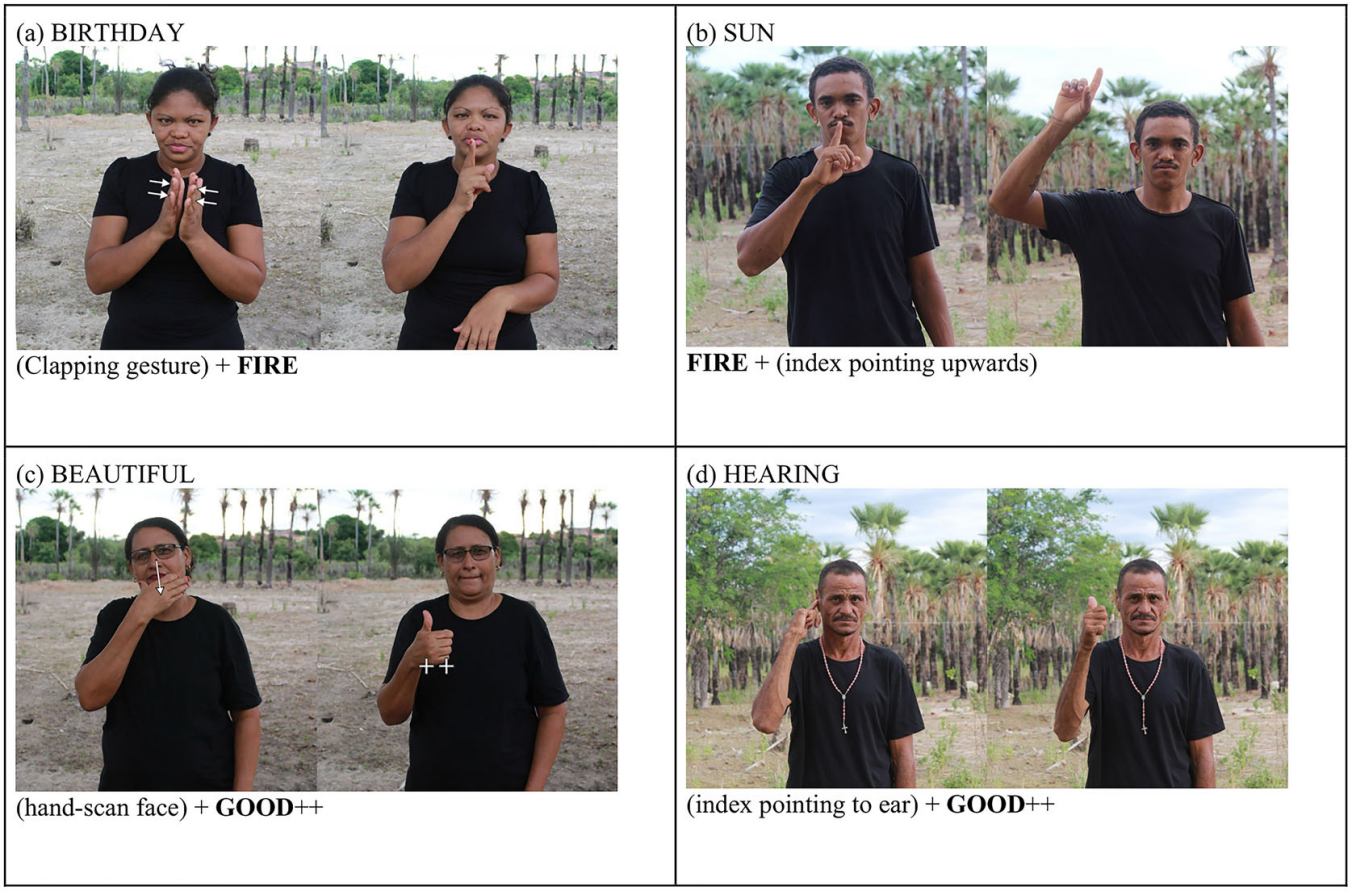
Samples of linguistic productivity in Cena compounding.

A critical component of any language is the ability to combine elements, such as words, to create new expressions, such as *sea‐side* and *light‐bulb*. This process requires a stable set of lexical items that denote types of entities, as well as the ability to combine these items in ways that generate new meanings. This linguistic capacity is a form of productivity that substantially enhances the expressive power of a language. One example of productive combination in Cena is the formation of compounds, where two meaningful items are combined to create new meanings. For instance, the sign for “fire” combines with a clapping gesture to create a compound meaning “birthday” (Fig. 2a) and with a finger pointing upward to create the compound for “sun” (Fig. 2b). The sign for “good” combines with hand scanning face to create the compound meaning “beautiful” (Fig. 2c) and with an index finger pointing to the ear to create the compound for “hearing” (Fig. 2d; see Almeida‐Silva & Nevins, 2020, for discussion). These are typical processes in sign language compounding, where signs are combined to create new, complex signs with novel meanings (Liddell & Johnson, 1989; Sandler, Bosch, Need, & Schiller, 1987).

**Fig. 3:**
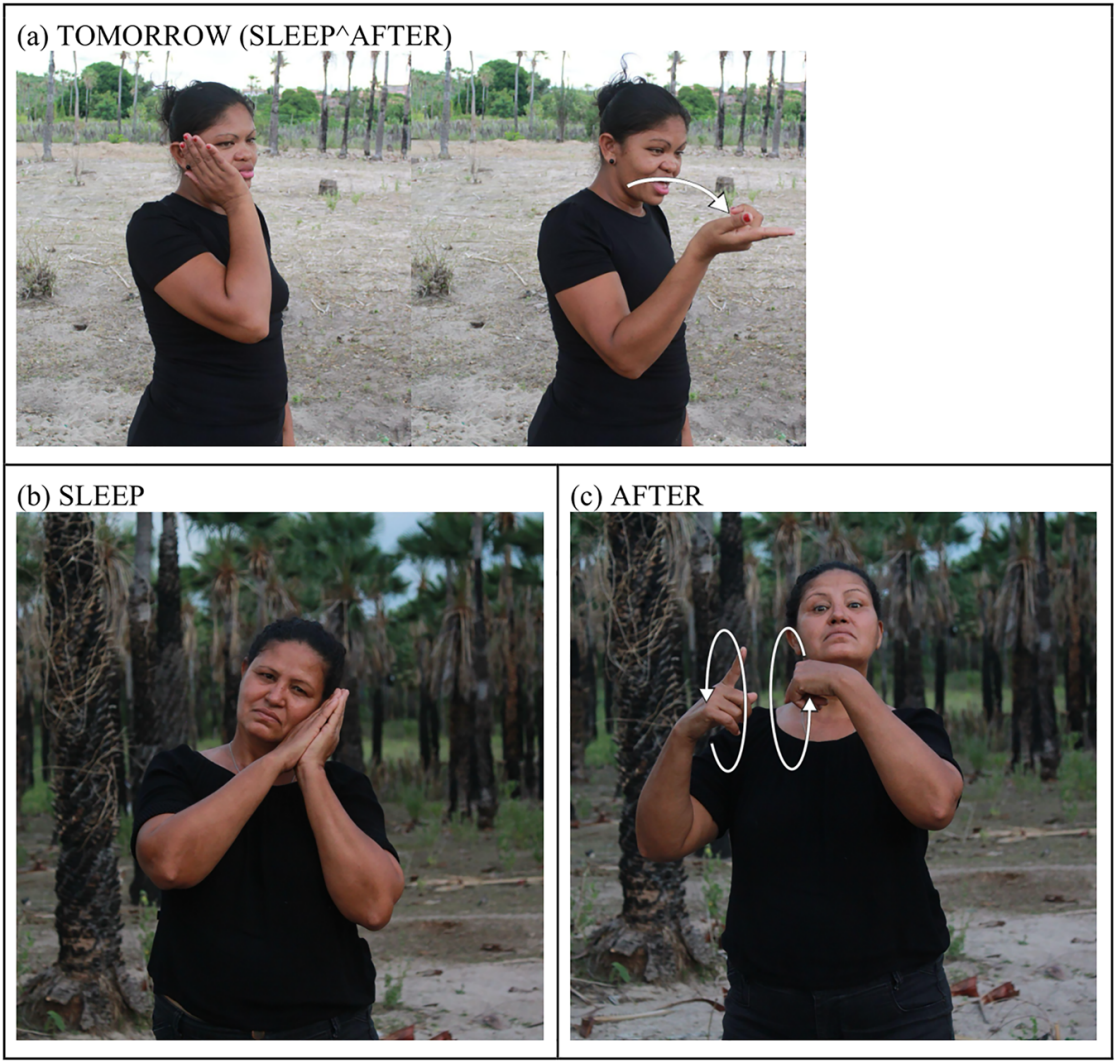
The compound word TOMORROW and its constituent members.

Other examples of productivity through compounding include signs like “tomorrow” (Fig. 3a), which combines the signs “sleep” (Fig. 3b) and “after” (Fig. 3c). It is interesting to note that while both “sleep” and “after” are bimanual signs requiring both hands, in the complex sign for “tomorrow,” they are simplified into one‐handed forms, likely to facilitate their combination into a single sign. Additionally, the bimanual circular motion in “after” is reduced to a single circular stroke in “tomorrow,” and the non‐manual facial expression of raised eyebrows in “after” is omitted in the compound.

### 6.3 Linguistic productivity: Complex lexical formation

**Fig. 4:**
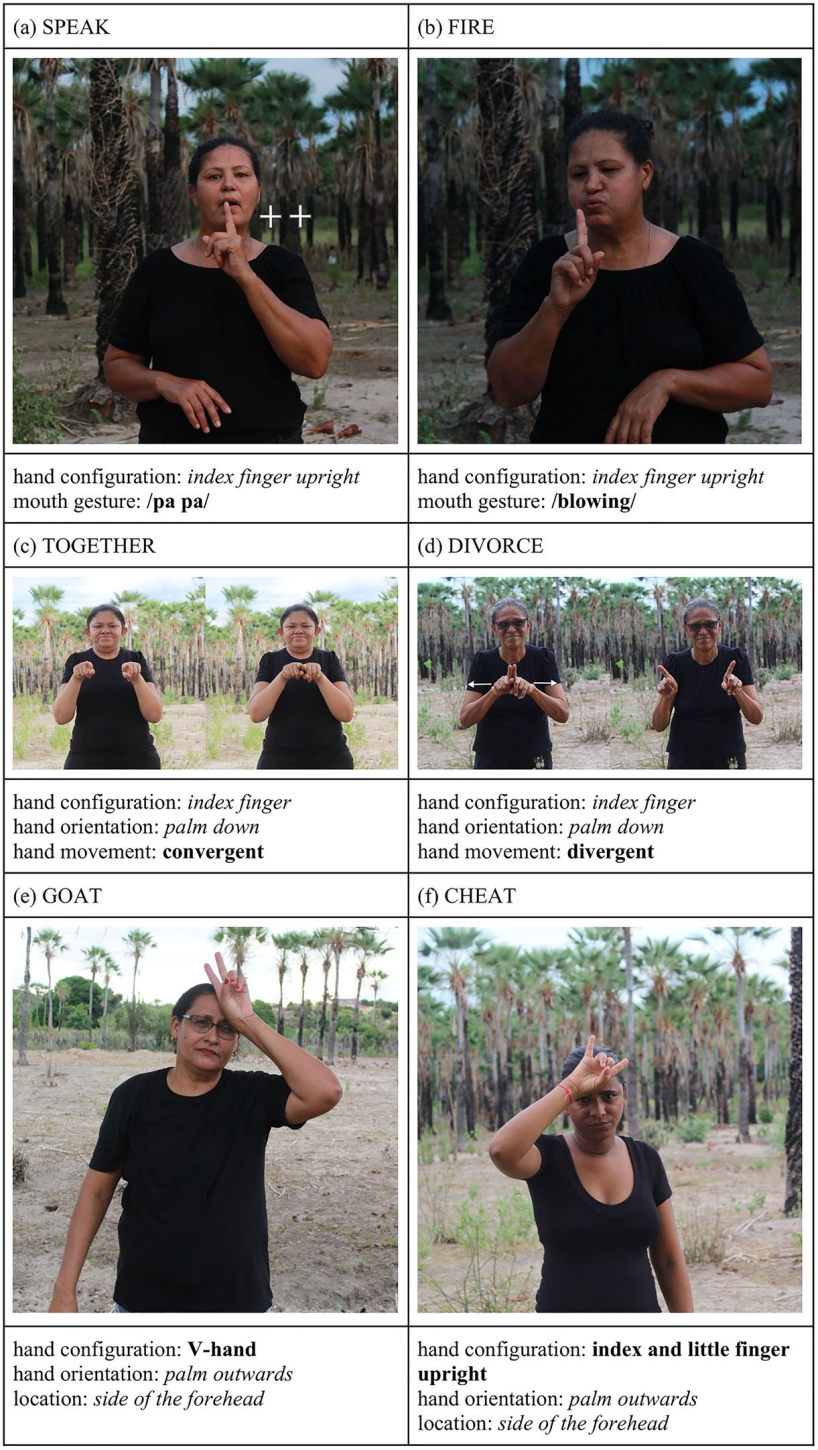
Minimal pairs in Cena. Each row displays a pair, such that the consistent feature is marked in italics and the contrasting feature, in bold letters.

In the compounds we examined, both members of the compound carry independent meaning, and the combination results in a composite meaning. However, there is another form of productivity commonly found in language that creates complex lexical items where one or both of the combined elements lack independent meaning, yet together they form a meaningful unit. For example, the element /comp‐/ in modern English, which has no meaning on its own, combines with various elements to form words like *comp‐atible*, *comp‐lete*, and *comp‐lex*. We find numerous examples of this type of complex lexical formation in Cena. For instance, the gesture of an index finger placed in front of the mouth, which by itself does not carry independent meaning, combines with lips mouthing a /pa pa/ shape to create the lexical term for “speak” (Fig. 4a). Similarly, combining an upward index finger with a blowing motion creates the lexical item for “fire” (Fig. 4b). Another example involves two index fingers pointing away from the signer, which by itself is meaningless, but when brought close together, it forms the lexical item for “together” (Fig. 4c), and when separated, it creates the lexical item “divorce” (Fig. 4d). Additionally, when the index and middle fingers are held in a V‐shape, which does not denote any meaning, and is raised in front of the forehead, it creates the lexical item for “goat” (Fig. 4e), while raising the index and little fingers in a similar manner signifies “cheat” (Fig. 4f).

### 6.4 Complex grammatical structure: The noun phrase

The nominal constituents that occupy subject and object positions often have their own internal structure. The central noun in these constituents, referred to as the head noun (e.g., CHILDREN, FARM, MEAT, TREE), can be modified by elements such as numerals (e.g., CHILDREN FOUR “four children”), possessive pronouns (e.g., MY FARM “my farm”), adjectives (e.g., MEAT FRIED “fried meat”), and quantifiers (e.g., TREE MANY “many trees”). This indicates the creation of phrases, which constitute a unitary constituent made up from two or more words, with a compositional meaning. For example, in Cena, it is possible to add an additional lexical item to a two‐term phrase (e.g., MILK HANDFUL THREE “three handfuls of milk”). Here, an already modified nominal constituent (MILK HANDFUL “a handful of milk”) is further modified by a numeral, a typical property of syntax at work.

**Fig. 5:**
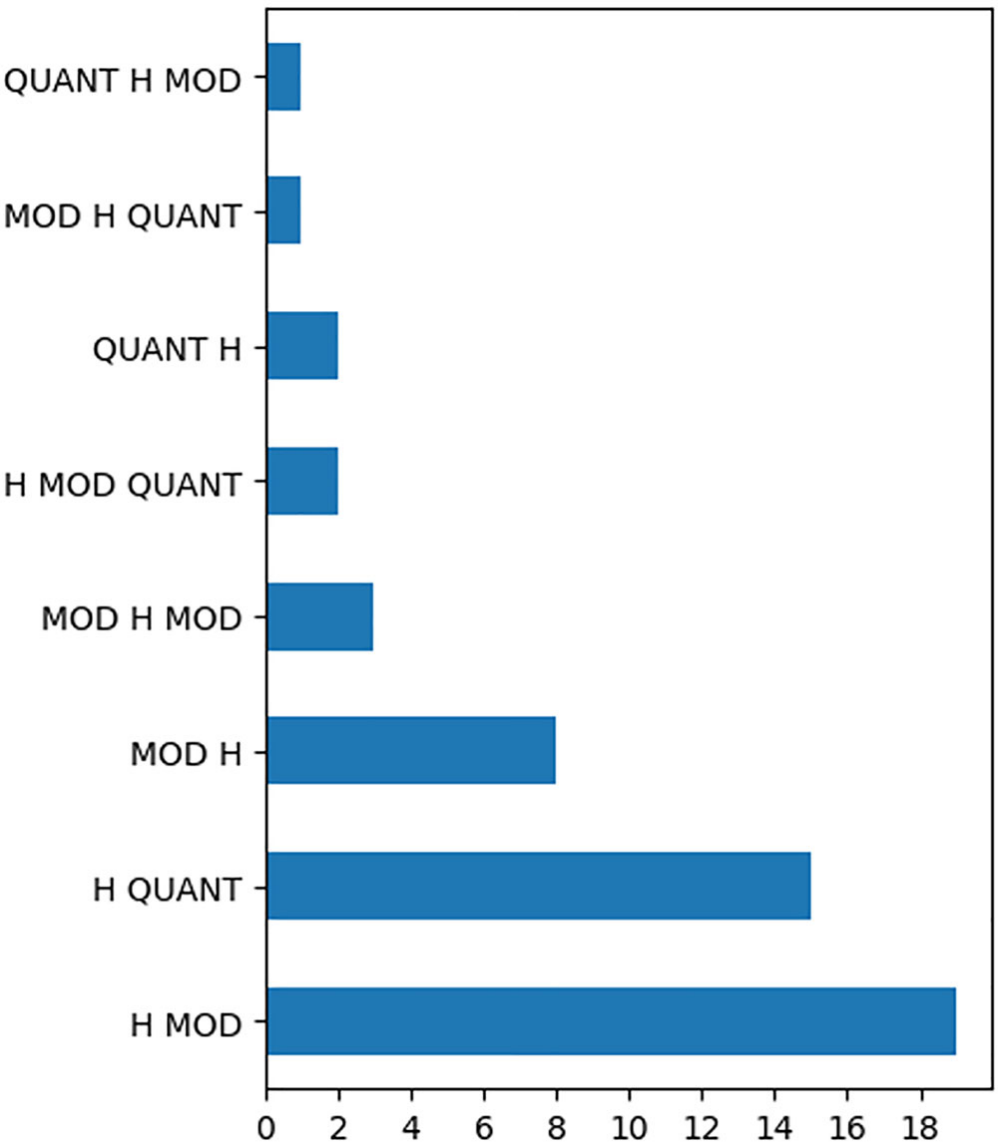
Frequency of Cena complex nominal constituent types. H: The head noun of the constituent. MOD: A modifying element. This can be an adjective, verb, or another noun. QUANT: A quantificational element. This can be a numeral or a quantifier such as any or all.

In order to show that complex nominal constituents are regularly present in Cena, we analyzed 188 noun phrases across 154 utterances from the Cena dictionary (Almeida‐Silva et al., 2023). These utterances were naturalistic utterances drawn from dialogues, local stories, and other narratives, see the Data section for details. The glossing was produced by researchers who are familiar with Cena and Libras. For each noun phrase (i.e., nominal constituent), we annotated the class of dependent elements in the phrase. For instance, the phrase MILK THREE would be annotated as “H QUANT,” representing a head noun followed by a dependent element, in this case, a quantifier. A brief description of the glossing terms is provided in Fig. 5, where we plot the distribution of all complex nominal constituents. Our analysis reveals that out of 188 noun phrases in the data, 51 are complex nominals, yielding a ratio of 27% of complex noun phrases.

To establish a comparable spoken language baseline, we conducted a similar (albeit automated) analysis on the spoken components of the British National Corpus (Love, Dembry, Hardie, Brezina, & McEnery, 2022), which contains naturalistic conversations. Here, we find a ratio of 32% of complex noun phrases over a total of 1,960,609 detected noun phrases (for this analysis, we discounted determiners, such as “*the*,” as CENA does not display overt determiners). This indicates that nominal modification is common in Cena and allows us to draw some conclusions about the structure of complex nominal constituents in the language. As shown in Fig. 5, Cena exhibits a preference for head‐initial nominal construction with two elements. While head final structures do occur, they are relatively rare in comparison.

## 7 Social Interaction as the key to language development

To reconstruct the development of Cena from its inception, we conducted interviews with four of the oldest residents of the village who interacted with and observed the first signer generations. Our goal was to reconstruct the history of the genesis of Cena on the basis of the memories of elders of the community who interacted with the first deaf people in VQ. Since there is no photographic or video record of the first deaf people born between the 1930s and 1960s, we have to rely on eyewitness accounts.

We created a semi‐structured questionnaire (which can be found in the Supporting Information) containing questions about the behavior of the first signers in the community. This includes examples of early signs and sign creation as well as details on how children interacted with each other. Further, we sought to confirm that the Cena language originated in complete isolation and without contact to other sign languages such as Libras. The interviews lasted between 40 and 60 min and were conducted by Anderson Almeida‐Silva. The interviews are transcribed in Portuguese in the Supporting Information to this publication.

**Fig. 6:**
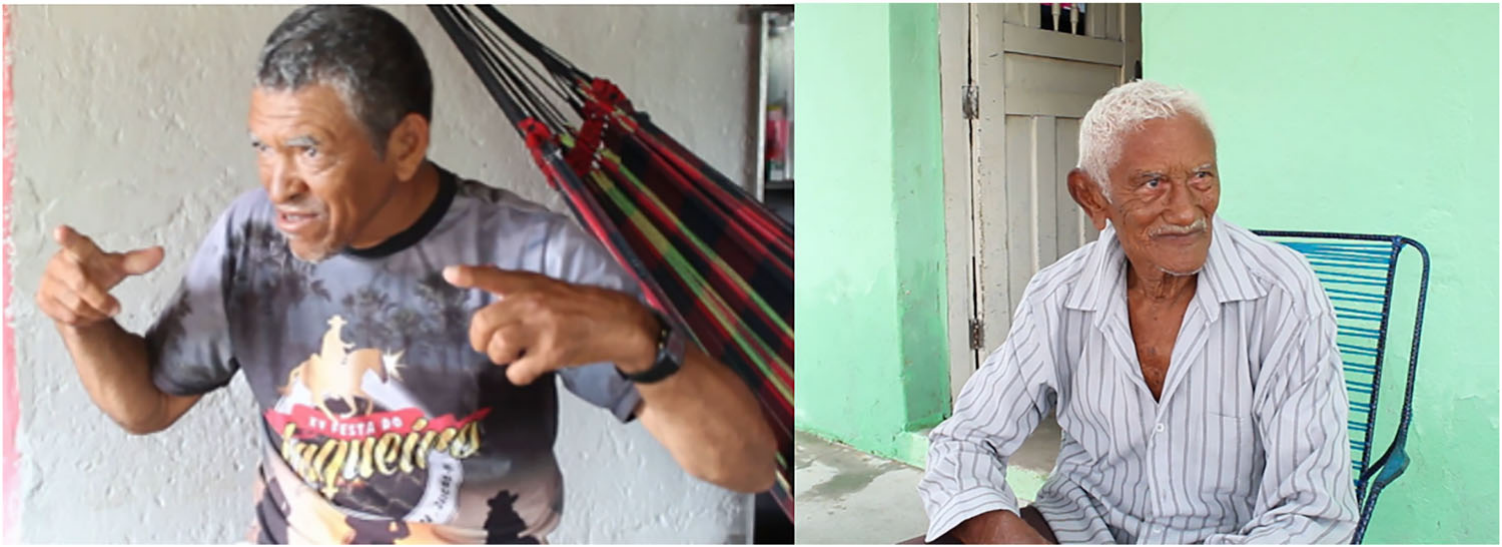
Interviews with Mr. Carvalho (left) and Mr. Edmundo (right).

The interviews were done under the permission of the Brazilian National Ethical Committee (*Plataforma Brasil*—Ministry of Health), registered with the authorization number CAAE–26198719.4.0000.0121. The interviewees consented to sharing their names and images with the scientific community. These are: (i) José de Sousa (78 years old), adoptive brother of the first deaf person born in the community, Pedro Cícero (born in 1932, hereafter PC); (ii) Luís (87 years old), a VQ resident and retired social worker; (iii) Edmundo (86 years old), uncle of deaf individuals from the second generation; and (iv) Alexandre Carvalho (60 years old), brother to five deaf individuals from the second generation, father to three deaf children from the third generation, and grandfather to one deaf person from the fourth generation. The complete questionnaire and the transcribed responses can be found in the Supporting Information. Fig. 6 shows the pictures of two of our interviewees.

What we can learn from these interviews is that Cena only became a fully fledged emergent language when the deaf were able to interact frequently with each other. When the first deaf person, PC, was born in 1932, there were no other deaf individuals in the community, and no one in VQ knew any sign language. Mr. de Sousa, PC's adoptive brother, recounted that as a child, he played with PC and that PC made efforts to communicate with hearing interlocutors, attempting to be understood. He mentioned that PC often used vocalizations to engage with hearing people, though there are few reports on PC's signs. Since PC had no other deaf (or otherwise signing) interlocuters during his early years, it is likely that his signing system did not develop beyond what we would expect for a homesign system.

**Fig. 7:**
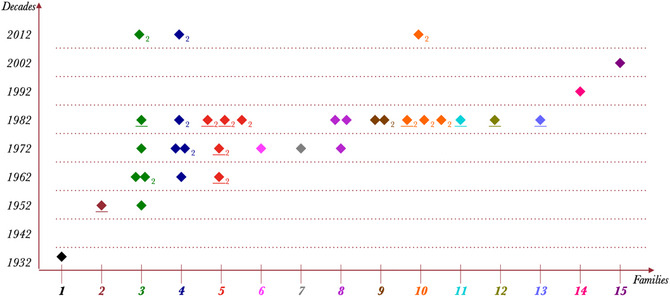
Demographics of Cena speakers. Each caret indicates a deaf child. The y‐axis tracks the decade of birth, and the x‐axis tracks different families. Each family is also coded as a distinct color. Underlined icons indicate the individuals who left the VQ community. Icons with a Number 2 index indicate individuals who developed a degree of fluency in Libras later in life (there are no Libras native signers in the village). The brown caret in 1952 is Lidia. The green caret next to Lidia was born in 1961.

When PC was 20 years old, Lidia, the second deaf individual in the community, was born. However, it took several years before Lidia was old enough to start engaging in signing conversations. Initially, she was isolated from PC due to a social taboo in VQ that prohibited boys and girls from playing together. She also remained isolated from the later generations of signers for some time due to a 10‐year age gap, as shown in the demographic chart of all deaf members of VQ in Fig. 7.

The 1960s–70s saw the birth of a larger number of deaf children in VQ, leading to the formation of a strong deaf community. Deaf individuals reportedly interacted with one another during religious festivities and other social activities in the village. This likely marks a period of rapid development for Cena as it evolved into an emergent sign system. The interviewees noted that Cena spread with the active involvement of hearing interlocutors, particularly adult caretakers who became part of this generation of signers. After this point, even more deaf children entered the community, as shown in the graph above.

An example of the lack of ambient sign language in the early stages of Cena is given by one of our interviewees, Mr. Carvalho. He recalls that his mother, who gave birth to five deaf children, did not know any sign language.

**Fig. 8:**
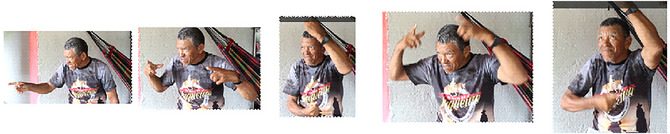
Mr. Carvalho performing a vivid memory of one of his children requesting an umbrella, before the conventional sign had come into existence.

Mr. Carvalho, who has three deaf children and one deaf grandchild, offers a particularly insightful recollection of how Cena developed into a fully functional language as more deaf children began interacting with each other. He recalls that the young deaf children, even during their crawling phase, initiated the process of signing by using body touch to call someone's attention and pointing toward the things they wanted. As they gained full use of their hands and facial muscles used for articulation, they began depicting objects and creating what would eventually become stable signs. He emphasized that his deaf children, along with others, were solely responsible for the creation and transmission of these signs, making them the principal drivers of language innovation in Cena. Mr. Carvalho also recounted witnessing one of his deaf children create the sign for “umbrella” (see Fig. 8). At the time, there was no established sign for umbrella, and the sign developed that day has since become the root of the modern Cena sign for umbrella.

## 8 Concluding remarks

Language emergence *de novo* is a rare event, albeit not as rare as previously assumed when we consider the many cases of emergent sign languages recently reported (Kastner et al., 2014; Meir et al., 2012; Nonaka, 2007; Kegl et al., 1999; Sandler et al., 2014). We argue that Cena is a particularly interesting example of this phenomenon, as we have access to community members who have been able to observe the entire history of the language up to the present day. Through them, we know that Cena has developed in complete linguistic isolation up until at least the 1990s. We can also assume, based on the eyewitness accounts, that Cena arose from the homesigns of the initial generations of deaf children born in the village.

We find that Cena and other languages like it have implications for theories of language acquisition. The existence of homesign suggests some form of innate linguistic capacity, as homesigns already show many properties of other natural human languages (Goldin‐Meadow & Yang, 2017; Goldin‐Meadow, 2005; Hunsicker & Goldin‐Meadow 2012; Goldin‐Meadow et al., 1995; Flaherty et al. 2021). This speaks in favor of a nativist account of language acquisition. At the same time, the history of Cena also shows that interaction between signers was critical in driving language development and acquisition. The first signer in the village was born two decades before the next deaf generation and was thus only able to interact with hearing community members during his acquisition period. Community members who remember him attest that some of his signs may be the basis of modern Cena signs but that his signs were limited, compared to those of later generations. The interviews further show that deaf signers were the main drivers of language development, such as new signs, and that those innovations arose from interactions between individuals. We argue that this speaks in favor of the interactionist theory of language acquisition. Both the interactionist and the nativist account can explain parts of the history of Cena, but to adequately describe Cena and its history, both views are necessary.

A language needs a community, and in the cases of emergent sign languages, that community is provided, be it through high incidence of deafness or through institutions. A community provides social interaction, which is critical for language acquisition (Adamson, 2018; Bloom, 2000; Bruner, 1981; Tomasello, 1992; Levinson, 2019). This interaction triggers the emergence of properties characteristic of human language in general. As more and more emerging sign languages are discovered, we will have more opportunities to understand what shapes the early development of a language.

## Funding

Vitor Nóbrega and Shigeru Miyagawa are funded by the São Paulo Research Foundation (FAPESP), Grant Numbers 2018/18900‐1 and 2023/03196‐5, respectively.

## Conflicts of Interest Statement

The authors declare no conflicts of interest.

## Data analysis

For the comparative analysis with spoken English, we used a spacy chunking pipeline and the spoken part of the British National Corpus (Love et al., 2017). All analyses of Cena data were done manually based on data derived from Almeida‐Silva and Araújo (2023). The interview data were produced by Anderson Almeida‐Silva.

## Data Availability

Dictionary data are available in Almeida‐Silva and Araújo (2023). Cena nominal data are available in the Supporting Information. Code necessary to replicate the analysis on the BNC is also available in the Supporting Information. Interview transcripts are available in the Supporting Information. Our Supporting Information can be found in the associated OSF storage: https://osf.io/qbrc2/overview?view_only=269023cbd32342648c0c8a9a73d68370.
